# Monitoring of *Clostridium botulinum* in Poultry and Cattle Manure During Spreading Operations

**DOI:** 10.3390/toxins18100406

**Published:** 2026-09-22

**Authors:** Rozenn Souillard, Sandra Rouxel, Virginie Dorenlor, Grégoire Kuntz, Félix Mahé, Typhaine Poezevara, Florent Eono, Amandine Avouac, Stéphane Kerphérique, Léa Jambou, Eloïse Cailton, Mathys Gouet, Sébastien Solanas, Loïc Balaine, Adeline Huneau-Salaün, Marianne Chemaly, Sophie Le Bouquin, Alain Hartmann, Caroline Le Maréchal

**Affiliations:** 1 Epidemiology Health and Welfare Unit, French Agency for Food, Environmental and Occupational Health & Safety (ANSES), 22 440 Ploufragan, France; virginie.dorenlor@anses.fr (V.D.); florent.eono@anses.fr (F.E.); stephane.kerpherique@anses.fr (S.K.); adeline.huneau@anses.fr (A.H.-S.); sophie.lebouquin-leneveu@anses.fr (S.L.B.); 2 Hygiene and Quality of Poultry and Pig Products Unit, French Agency for Food, Environmental and Occupational Health & Safety (ANSES), 22 440 Ploufragan, France; sandra.rouxel@anses.fr (S.R.); typhaine.poezevara@anses.fr (T.P.); marianne.chemaly@anses.fr (M.C.); caroline.lemarechal@anses.fr (C.L.M.); 3 INNOVAL, Brittany Animal Health Defense Group, 22 440 Ploufragan, France; gregoire.kuntz@innoval.com (G.K.);; 4 Agroecology Research Unit, French National Research Institute for Agriculture, Food and Environment (INRAE) and Burgundy Europe University (UBE), 21 065 Dijon, France; sebastien.solanas@inrae.fr (S.S.); alain.hartmann@inrae.fr (A.H.)

**Keywords:** botulism, animal, soil, manure

## Abstract

Animal botulism mainly affects poultry and cattle, with risks of cross-contamination. This study aimed to evaluate *Clostridium botulinum* contamination in manure and soil following manure spreading on 13 clinically affected farms (10 cattle and three poultry) and 39 clinically unaffected farms (20 cattle and five poultry farms without any previous botulism outbreak, six poultry farms with a history of botulism and eight mixed poultry/cattle farms with an ongoing bovine botulism outbreak). Twelve manure samples, two swabs on a manure pile, 12 soil samples, and two boot swabs from fields were collected before manure spreading. Twelve soil samples and two boot swabs from fields were obtained after manure spreading. *C. botulinum* types C, D, C/D, and D/C were detected using real-time PCR after an enrichment step. On clinically unaffected poultry and cattle farms with no history of botulism, *C. botulinum* was detected in 1.4% and 1.8% of samples, respectively. Detection rates reached 8% on poultry farms with a history of botulism and 42.8% on poultry farms with ongoing bovine outbreaks. On clinically affected poultry and cattle farms, 34% and 18.9% of samples were positive, respectively. This study highlights the presence of *C. botulinum* in manure and soil and emphasizes the importance of biosecurity measures to prevent cross-contamination and disease recurrence.

## 1. Introduction

Botulism is a neurological disease due to the action of botulinum neurotoxins (BoNT) that inhibit the release of acetylcholine in the nerve terminals, inducing a flaccid paralysis. These toxins are produced by clostridia, in particular *Clostridium botulinum*, a spore-forming bacterium that is highly resistant to adverse conditions and able to survive for years in the environment [1,2,3]. Human botulism is mostly associated with BoNT-A, B, E, and F, while animal botulism is mainly due to BoNT-C, D, and mosaic C/D and D/C [4,5]. Avian botulism is mostly due to BoNT-C/D, while cattle botulism is mostly due to BoNT-D/C [4].

In France, cattle and poultry farms are affected by botulism every year [4]. Between 2009 and 2019, an average of 30 outbreaks were diagnosed each year on poultry farms and 10 outbreaks on cattle farms [4]. Broilers have been shown to be asymptomatic carriers of *C. botulinum* type D/C and to be able to induce outbreaks in cattle, notably due to biosecurity failures [6]. Poultry manure has, in particular, been highlighted several times in the literature as a potential source for initiating outbreaks of cattle botulism [6,7,8,9,10,11]. While the presence of *C. botulinum* in poultry manure after a clinical botulism outbreak has been reported, its frequency in manure without an avian botulism outbreak is unknown [12]. In the same way, field investigation of farms affected by cattle botulism and farms that were not affected, both before and after manure spreading, has never been previously conducted. Overall, the available scientific literature suggests that manure may be contaminated with *C. botulinum*, highlighting the importance of appropriate manure management practices to prevent the transfer of contamination to other animal species, farms, and, more broadly, the environment. Such measures could contribute to reducing the risk of initiating new botulism outbreaks and limiting disease recurrence. Several strategies for managing contaminated manure have been proposed, including its destruction by incineration [13,14]. However, this radical approach presents several limitations, particularly in terms of operational feasibility, and raises concerns from both agronomic and environmental perspectives. In the context of a clinical animal botulism outbreak, implementing a specific manure management plan may nevertheless be required, as contamination of the farm and its manure can reasonably be suspected, even though the actual level of contamination remains unknown.

Among the alternative manure management strategies, land application may be considered. Soil is indeed recognized as an environmental reservoir for clostridia, including *C. botulinum* [15]. Consequently, applying *C. botulinum*-contaminated manure to soils that already harbor this bacterium could potentially represent a viable management option. However, assessing the relevance and safety of this practice requires characterization of the initial level of soil contamination, as well as an understanding of the fate and persistence of *C. botulinum* types C, D, C/D, and D/C following manure application. In particular, the introduction of *C. botulinum* types C, D, C/D, and D/C into agricultural soils could result in widespread contamination at the field level and potentially increase animal exposure through feed production, including grazing, hay, and silage.

The persistence of *C. botulinum* in agricultural soils following the application of contaminated organic material has already been experimentally demonstrated. Gessler and Böhnel (2006), for example, reported persistent contamination of soil after the application of compost contaminated with *C. botulinum* type D, suggesting that the bacterium may remain viable in fields over extended periods [16]. In addition, soil particles can adhere to forage plants, resulting in the incidental ingestion of soil by livestock consuming contaminated forage [17]. In silage, insufficiently acidic conditions may further create an environment conducive to bacterial growth and botulinum toxin production [18,19]. Moreover, *C. botulinum* type D has been shown to be internalized by clover plants grown in contaminated soil, suggesting that contaminated soils may facilitate the transfer of the bacterium to crops intended for animal feed [20]. Given the ability of *C. botulinum* to form spores that are capable of persisting for long periods in the environment and its potential transfer from contaminated soil to animals through feed production, better understanding of the fate of *C. botulinum* types C, D, C/D, and D/C in soil following manure application is needed. This may contribute to determining whether land application can be considered a safe and appropriate manure management strategy. It is also particularly relevant in situations involving undetected or asymptomatic manure contamination, for which the potential consequences of land application on soil contamination are currently difficult to assess.

In this context, the objectives of this study were to evaluate the contamination of poultry and cattle manure by *C. botulinum* either from farms after a clinical botulism outbreak or without any clinical signs, as well as to evaluate soil contamination after application of manure on fields.

## 2. Results

### 2.1. Description of Poultry and Cattle Farms and Manure Management

A total of 52 farms were investigated in the study, including 22 poultry farms and 30 cattle farms. The twenty-two poultry farms were divided up into four categories (Table 1). Three farms where clinical signs were observed in poultry (i.e., three ongoing avian botulism outbreaks) formed the first category. The remaining 19 farms, with no clinical signs in poultry, were divided up into three categories: five farms with no history of botulism, six farms that had already had an avian or cattle botulism outbreak within the last 4 years, and eight farms with an ongoing clinical bovine botulism outbreak. For the 19 clinically unaffected poultry farms, ten broiler flocks (average size, 23,052 animals, ranging from 6528 to 30,600 animals), eight turkey flocks (average size, 10,049 animals, ranging from 7200 to 16,480 animals) and one laying hen flock (missing data for the size) were investigated. All three poultry farms with an ongoing botulism outbreak (2 C/D and 1 D/C) produced turkeys (Supplementary Data S1).

Among the 30 cattle farms, 20 farms were clinically unaffected and 10 were affected by botulism (Table 2). None of the enrolled farms had a history of botulism. The 20 clinically unaffected cattle farms included 16 dairy farms, three farms with dairy and beef cows, and one with only beef cows. Two of them were mixed farms with poultry as well. Eight of the 10 bovine botulism outbreaks were on dairy farms and two were on farms with both dairy and beef cows. Eight were affected by botulism type D/C, one by type C, and for one, the BoNT type was unknown (i.e., missing data) (Supplementary Data S2). Seven of the 10 botulism-affected cattle farms were mixed farms with poultry flocks. For four of them, the clinically unaffected poultry flock on the farm was also investigated in our study as a poultry farm with an ongoing bovine botulism outbreak (Supplementary Data S2).

Regarding manure management on the clinically unaffected poultry and cattle farms (Table 1 and Table 2), most of the farmers stored the manure for several weeks before spreading and burying it in the fields. On two of the three botulism-affected poultry farms, manure was spread directly over the field without any storage, whereas on the third farm, the manure was stored for three months before spreading. All of them buried the manure as it was being spread. In most of the botulism-affected bovine farms, the manure was stored for several months. Three of the farms added quicklime to the manure, which was buried during spreading. Poultry and/or bovine and/or pig manure had been applied in most of the fields in the previous two years (Supplementary Data S1 and S2), and the fields had mainly been used for growing cereal crops.

### 2.2. Detection of Clostridium Botulinum on Poultry Farms

*C. botulinum* was detected in the manure and soil in the four categories of poultry farms investigated in the study (Figure 1 and Supplementary Data S3).

For the five clinically unaffected poultry farms without any history of botulism (Figure 1 and Supplementary Data S3a), *C. botulinum* was detected in only 1.4% of the environmental samples collected (3/209 samples) on three farms, including two positive samples in manure (2.8%) and one positive sample (1.4%) in soil before manure spreading. It was only detected once on one of these five farms via cloacal swabs.

On clinically unaffected poultry farms with a known history of botulism (Figure 1 and Supplementary Data S3b), *C. botulinum* was detected in 8% of all environmental samples (18/224 samples) on three farms out of six, with a higher detection rate in manure (13% of positive samples) than in soil (5.7% and 4.3%, respectively, before and after manure spreading). It was not detected in any cloacal swabs from the poultry.

The highest detection rate in clinically unaffected poultry flocks was obtained from flocks on mixed farms with an ongoing bovine botulism outbreak (Figure 1 and Supplementary Data S3c), with 42.8% of positive environmental samples on six out of eight farms. *C. botulinum* type D/C was the most common type detected in samples from these clinically unaffected poultry flocks (82.4%). The *C. botulinum* type detected in poultry was the same as the one detected in botulism-affected cattle on these six farms. Manure was the most contaminated matrix, with 65.3% of positive samples. *C. botulinum* was also detected in soil, with 17.1% and 38.1% positive samples, respectively, before and after manure spreading. It was possible to collect cloacal swabs on three poultry farms in this category, with a detection rate of 0% (0/20), 15% (3/20), and 55% (11/20).

On the three botulism-affected poultry farms (Figure 1 and Supplementary Data S3d), *C. botulinum* was detected in 34% of manure and soil samples (34/100 samples), with a high detection rate in manure (56.7% of positive samples) and a lower rate in soil (17.8% and 28.6%, respectively, before and after spreading). Cloacal swabs were collected on two farms, with a detection rate of 25% and 75%.

For four poultry farms where soil samples collected after spreading were positive, extra soil samples were collected again several months after spreading. Of these four farms, two were clinically unaffected poultry farms with an ongoing D/C bovine outbreak. Soil samples were collected six months after the spreading of contaminated manure on one farm and one year after on the other. On the first farm, *C. botulinum* was still detected in one sample (out of 12) six months after manure spreading. On the second farm, *C. botulinum* was still detected one year after spreading (one positive sample out of 12). Regarding the two remaining farms, which were both botulism-affected poultry farms, *C. botulinum* was no longer detected six months after spreading in one case but was still frequently detected six months after spreading in the other one (all 12 soil samples were positive).

### 2.3. Detection of Clostridium Botulinum on Cattle Farms

*C. botulinum* was detected in manure and soil in the two categories of cattle farms investigated (Figure 1 and Supplementary Data S4). Regarding clinically unaffected farms with no history of botulism (Figure 1 and Supplementary Data S4a), *C. botulinum* was detected in only 1.8% of all the samples collected (15/840) from five farms out of the 20, mostly in soil. For the 10 botulism-affected farms (Figure 1 and Supplementary Data S4b), *C. botulinum* was detected in 18.9% (64/338 samples) of all the samples collected from 6/10 farms, with a high detection rate in manure (32.8% of positive samples from five farms) but also detection in the soil (9.5% and 11.1% before and after spreading, respectively).

Extra soil samples were collected either six or 12 months after spreading from the field of two cattle farms with positive soil samples just after spreading. On the first farm, *C. botulinum* was detected in one soil sample out of the 12 collected in the field six months after manure spreading. For the second farm, *C. botulinum* was detected in seven soil samples out of the 12 collected one year after the spreading of contaminated manure.

### 2.4. Factors Influencing Clostridium Botulinum Detection in Manure and Soil Samples

Figure 2 shows the results of the mixed logistic regression model for the risk of detecting *C. botulinum* in manure and in soil samples before spreading and after spreading. The risk of detecting *C. botulinum* was higher for manure originated from poultry than from cattle farms (odds ratio, 32.2; confidence interval, 95% [2.54–408]), for swab samples than for flask samples (OR = 3.00, [1.12–8.02]) and when a clinical botulism outbreak, affecting bovine or poultry, was ongoing on the farm (OR = 439, [29.4–6558] and OR = 307, [3.13–30,121] for bovine and poultry respectively). On the contrary, none of these factors influenced the risk of *C. botulinum* detection in soil samples before manure spreading. In particular, the risk of detecting a positive soil sample before manure spreading was not significantly higher on poultry farms than bovine farms (OR = 5.32, [0.66–43.1]). Similarly, past outbreaks of botulism on poultry farms did influence the risk of detecting *C. botulinum* in soil samples before manure spreading (OR = 0.36 [0.05-2.85])). None of the factors studied showed a significant effect on soil contamination before spreading.

The soil contamination before manure spreading was considered in the model for the risk of *C. botulinum* detection in soil samples after spreading. The risk of detecting *C. botulinum* in soil samples after spreading was higher on poultry farms than bovine farms (OR= 15.8, [1.38–181]) and increased with the number of positive soil samples found before spreading (OR = 3.66, [2.25–5.95] for each supplementary positive sample). The number of manure samples detected positive for *C. botulinum* was not introduced in the model, as this parameter was highly correlated with the type of farm. This observation suggests that the risk of soil contamination after spreading depended both on the initial contamination of the soil before spreading and on the contamination of the manure applied, as poultry manure was more at risk of being contaminated by *C. botulinum* than bovine manure.

### 2.5. Physicochemical Parameters in Soil

Results of the textural and chemical analysis from 27 soil samples for which data were available are presented in Supplementary Data S5. Detection of *C. botulinum* C, D, C/D, or D/C (in 9/27 soil samples) was statistically associated with more acidic (average pH of 5.6 ± 0.3 in positive soils versus 6.6 ± 0.9 in negative soils, *p* < 0.01), dryer (residual humidity of 11.5 mg/kg ± 4.5 versus 17.5 mg/kg ± 6.6, *p* = 0.02) soils. Regarding chemical composition, soils positive for *C. botulinum* C, D, C/D, or D/C showed a lower concentration of available calcium (1.85 mg/kg ± 2.6 versus 9.6 mg/kg ± 8.5, *p* = 0.02) but higher concentrations of available phosphorus (0.13 mg/kg ± 0.06 versus 0.05 mg/kg ± 0.03, *p* = 0.001) and available manganese (0.04 ± 0.01 mg/kg versus 0.02 ± 0.01 mg/kg, *p* < 0.01). As a result, the average cationic exchange capacity of soils positive for *C. botulinum* C, D, C/D, or D/C was lower (7.3 cmol+/kg ± 2.4) than that of negative soils (12.9 cmol+/kg ± 7.0).

## 3. Discussion

Animal botulism mainly affects poultry and cattle in France [4], with a known risk of cross-contamination between the two sectors [21,22,23]. Poultry can be asymptomatic carriers of *C. botulinum*, and poultry manure has been reported to be a source of contamination in cattle outbreaks [6,7]; indeed, it is even suspected of being one of the main sources. It is known that manure from poultry farms affected by botulism is contaminated [12] and that *C. botulinum* can survive for many years in soil [1,2]. This study provides, for the first time, data on the detection of *C. botulinum* in manure and soil from poultry and cattle farms either affected or not by the disease, with monitoring of contamination under field conditions. *C. botulinum* was, in particular, detected on botulism-affected poultry farms (34% of environmental samples were positive), affected cattle farms (18.9%) and mixed poultry farms with an ongoing outbreak in cattle (42.8%), and—to a lesser extent—it was detected on poultry farms with a previous history of botulism (8%). Farms with no such history were the least contaminated (1.4% for poultry and 1.8% for cattle). However, they are not entirely free of the bacterium, suggesting its low-level presence even on farms with no history of botulism. Several hypotheses can be put forward to explain the presence of *C. botulinum* on these farms. *C. botulinum* types C, D, C/D, or D/C could be naturally present in the digestive tract of livestock (cattle [24], poultry [6], or other animals such as pigs [25]) at low levels, perhaps below the detection threshold of the methods currently available to us [26]. Contamination could result from airborne dissemination of spores during the spreading of manure by other farmers in the same area. Airborne dissemination has already been suggested for other clostridia such as *Clostridioides difficile* during the spreading of poultry manure [27]. Contamination could also originate from wildlife. In this study, *C. botulinum* type C, D, C/D, or D/C was detected in manure and soil from all farm categories, both in poultry and cattle. Only one liquid manure was included in our study to avoid multiplying the different matrices, although it can also be contaminated after an outbreak [6]. However, given current developments in dairy production systems, particularly milking robots, it would be interesting to study liquid manure contamination, establish sampling methods, and determine the fate of the bacterium in this matrix.

Here, a total of 52 farms were investigated, including five production types (broilers, turkeys, hens, dairy cows, and beef cows), clinically unaffected farms with or without a history of botulism, and botulism-affected farms. Farms were selected on a voluntary basis. It should be noted that some farmers did not agree to participate in our study. One of the explanations was the extremely difficult situation that farmers have to deal with when an outbreak occurs on a farm with high mortality levels, financial loss, and the unavailability of any control measure to stop the outbreak in the case of cattle botulism. The psychosocial impact of such an animal disease on farmers or veterinarians, for example—already studied in the context of the avian influenza crisis [28]—has been poorly characterized up to now for botulism despite the clear effects on them when facing an outbreak. Another explanation for the unwillingness of some farmers to participate in our study was their fear of the potential consequences of the detection of *C. botulinum* on their farm from an administrative perspective. Current French regulations do not provide fully explicit guidelines on the management of animal botulism, particularly concerning measures to be implemented following bacterial detection or during outbreak situations. Although not included in the European Animal Health Law, animal botulism is a notifiable disease in France [29]. However, there is currently no regulation describing the measures that should be applied in the event of detection of clinical signs or the bacterium itself, resulting in case-by-case management. The selection of farms in our study was also based on some criteria related to manure management practices, such as no mixing of various effluents and no exporting of manure outside the farm. Moreover, during the investigated period (2019–2023), a decrease in the number of analyses due to a suspicion of poultry botulism was observed in France. Whether this could be linked to a real decrease in outbreaks or to the regulation context and the reluctance of farmers to perform analyses is unknown. However, this impacted our study, as the number of botulism-affected poultry farms that could be included was extremely low. The farmers’ refusal to take part in the study might also have introduced a selection bias that is difficult to estimate due to the lack of information on manure practices on these farms. Given the diversity of manure management practices observed in our study, no statistical tests were applied to evaluate the potential links between the detection of *C. botulinum* and these practices.

In our study, different matrices were analyzed: manure and soil samples. Manure provided the highest number of positive samples, especially from botulism-affected poultry and cattle farms, and from clinically unaffected poultry farms with an ongoing outbreak in cattle. For the affected poultry farms, *C. botulinum* was frequently detected in manure on the three farms investigated (56.7% of positive samples), confirming previous results obtained from poultry manure collected after botulism outbreaks [12]. *C. botulinum* was detected in manure samples from more than half of the cattle farms after a clinical outbreak (with 32.8% of positive samples). These findings highlight the contamination of manure by *C. botulinum* after cattle botulism outbreaks and show that dedicated measures are required to manage such contaminated manure. However, the management of contaminated manure is difficult to implement on farms. Available studies show that neither prolonged storage, composting nor anaerobic digestion can completely sanitize effluents [12,30]. Treatment by alternating layers of manure and quicklime is recommended for contaminated manure. The advantage of this approach lies in the reduction in volume of effluent through combustion, but this method is risky and does not result in complete elimination of *C. botulinum* spores [12,30]. Given these difficulties, it is crucial to comply with good practices and biosecurity measures when managing contaminated manure on farms to avoid recurrence of the disease, cross-contamination between animal productions, and to identify the most suitable land for spreading based on its location and subsequent use for crops.

Despite a higher risk of detecting *C. botulinum* in manure from farms affected by clinical outbreaks, positive manure samples were also found on non-affected poultry farms with or without past outbreaks of botulism. The detection of *C. botulinum* in the manure of poultry farms with a known history of botulism suggests a silent presence of *C. botulinum* on farms that have already been affected by the disease. No typical clinical signs evocative of botulism had been reported in these flocks, suggesting long-term detection of spores in the environment and potential carriage by poultry without development of the disease. This is also suggested by the detection of *C. botulinum* D/C in manure and cloacal swabs collected from poultry farms with an ongoing cattle outbreak or even from poultry and cattle farms with no history of botulism. The asymptomatic carriage of *C. botulinum* type D/C by broilers was previously reported on a mixed farm [6] and appears to be confirmed here. The detection of *C. botulinum* C/D and *C. botulinum* D has also been reported previously [24]. Further investigations are now needed to determine the prevalence of this carriage, the risk factors and the mechanisms behind switching from asymptomatic carriage to clinical signs. This again highlights the crucial importance of biosecurity and sanitary barriers between productions when managing manure on mixed farms, even without a botulism outbreak.

*C. botulinum* was detected in soil before manure spreading in all categories of farm investigated, whatever their status regarding botulism outbreaks (ongoing or past outbreaks) or the type of farms (poultry or bovine). This result was surprising, as a recent study conducted to establish an overview of soil contamination in France showed a low rate of detection of *C. botulinum* type C, D, C/D, and D/C (1.7%) [31]. This may be due to the difference in the sampling plan or the location of soil samples collected all around France, in the other study, versus amended fields in regions with agricultural breeding activities here in the northwest of France. Indeed, poultry and/or cattle manure had been spread on the sampled field for at least the previous two years on most of the poultry and cattle farms investigated. It is likely that the manure spread over the investigated fields in previous years was contaminated given the potential asymptomatic carriage of *C. botulinum* by poultry and cattle [6]. However, other sources of soil contamination should also be considered, such as the carcasses of wild animals or asymptomatic carriage of *C. botulinum* by wildlife [32].

The different findings regarding *C. botulinum* detection here in amended fields versus all around France in the previous study [31] is also associated with the soil’s contrasting physicochemical characteristics. In the national soil survey, BoNT-B-producing clostridia were more frequently detected and their detection was mainly associated with higher pH, humidity, concentration of cationic minerals and cation-exchange capacity [31]. On the contrary, *C. botulinum* type C, D, C/D, and D/C were associated with low pH, humidity concentration of cationic minerals and cation-exchange capacity. The physiological characteristics of the different groups of *C. botulinum* (group I or II for *C. botulinum* strains producing BoNT-B and group III for those producing BoNT-C, D, C/D, D/C) are known to be different [33]. This may explain the differences between the contrasting profile of physicochemical characteristics of soil in which *C. botulinum* type B or C, D, C/D, and D/C are found.

*C. botulinum* was also detected in soil after manure spreading in all farm categories except poultry farms with no history of botulism. The risk of finding positive soil samples after spreading was higher on poultry farms than on cattle farms, where the manure was also more at risk to be positive for *C. botulinum*. These results suggest that spreading contaminated manure on land could lead to the long-term detection of *C. botulinum* in soil and, more globally, in the environment. Variability in the results of soil contamination after spreading may also be explained by multiple factors such as the initial level of soil contamination, the manure spreading method and equipment, or intrinsic biotic and abiotic soil factors such as physicochemical characteristics, soil microbial communities, or climatic conditions (rain, dryness, etc.). Such factors could impact either the growth or survival of *C. botulinum* or may influence the ability of the method applied here to detect *C. botulinum* in the soil.

In our study, we used different sampling strategies by using either swabs or boot swabs in addition to manure and soil samples Regarding the sampling of manure in piles, the frequency of *C. botulinum* detection was significantly higher when using swabs on the surface (25.5%, 25/98) than when using manure samples collected at a depth of 15 cm (18.9%, 112/592). This observation was confirmed by the higher risk of detection associated with swabbing than with flask sampling in the regression model. It had previously been reported that the surface of manure piles showed higher detection levels [12], which may explain this result. No statistical difference was observed in levels of *C. botulinum* detection in soil samples in flask (8.7%; 95/1091) than via boot swabs (5.5%, 10/182) before or after spreading. Boot swabs only sample the soil surface, which is potentially more exposed to climatic conditions such as leaching by rain or UV exposure. Conversely, soil samples were collected at a depth of 30 cm because all the farms buried their manure at this depth. Nonetheless, as no statistical difference was detected between boot swabs and soil samples, boot swabbing might be an alternative method to soil sampling, which remains labor- and time-consuming. To summarize, this study shows that swabs and boot swabs are relevant user-friendly collection methods appropriate to evaluate the contamination of *C. botulinum* in manure and soil samples.

This study shows that, regardless of the type of farm considered—including farms with no history of botulism and in the absence of clinical signs—it is possible to detect *C. botulinum* and that manure should be handled with caution all the time to prevent the bacteria from being released into the environment and animal exposure. Based on the results obtained here, manure from farms with an ongoing outbreak should not be spread on fields, especially on clinically affected poultry farms or in the case of asymptomatic carriage by poultry. This study also illustrates the importance of a global integrated approach when considering the management and prevention of the dissemination of contaminants in agriculture activities, by working not only at the farm level but also on a larger scale, by including fields used for crop production and not segmenting activities (livestock/crops) but considering them as a whole.

## 4. Conclusions

This study highlights, for the first time, the presence and fate of *C. botulinum* through application in fields of manure from poultry and cattle farms affected or not by botulism. These findings emphasize the critical role of biosecurity, in particular, as far as manure management is concerned, even in the absence of clinical outbreaks. Good manure management practices (keeping manure away from the animal production areas, cleaning spreading equipment and vehicles, no surface spreading of manure on plots, no cattle grazing on spread plots) and sanitary barriers between animal productions (compliance with hygiene locks for staff, disinfection of shared equipment, management of vehicle flows on the farm) are fundamental for preventing both the risks of cross-contamination by *C. botulinum* between the productions and recurrence of the disease. These biosecurity measures need to be enhanced on farms affected by botulism by a reinforced manure management plan. Since animals affected by botulism are paralyzed and immobile, isolating them in a confined area could limit the amount of contaminated bedding and thus indirectly limit the amount of contaminated manure that needs to be managed. This study shows that soil amended by poultry or cattle manure can be contaminated by *C. botulinum*. The potential transfer of this bacterium to crops and then to animals is still unknown and should now be explored to assess risks and propose suitable preventive measures [18,20].

## 5. Materials and Methods

### 5.1. Selection of Farms

This study was carried out in northwestern France between July 2020 and June 2023 and involved 22 poultry farms divided in 4 categories (five clinically unaffected poultry farms without any history of botulism, six clinically unaffected poultry farms with a history of botulism (within the last 4 years), eight clinically unaffected poultry farms on a mixed farm with an ongoing bovine outbreak and three affected poultry farms) and 30 bovine farms (20 clinically unaffected and 10 affected farms). The farms were selected on the basis of the farmers’ willingness to take part.

The 19 clinically unaffected poultry farms were selected after contacting the veterinarians of six production organizations. The 20 clinically unaffected bovine farms were selected by the association for herd disease control (Groupement de Défense Sanitaire—Innoval, Ploufragan, France) that provides technical support to cattle farmers and their veterinarians.

The outbreaks on the three botulism-affected poultry farms were reported by veterinarians after seeing typical signs such as flaccid paralysis and high mortality. Regarding the 10 affected bovine farms, veterinarians reported the outbreaks following the observation of several deaths in a short period of time and clinical signs (more or less obvious flaccid paralytic disease). All outbreaks were confirmed by analysis using samples collected from symptomatic animals (poultry or bovine) and analyzed by the French National Reference laboratory prior to their inclusion in this study.

### 5.2. Sample Collection and Epidemiological Data

Environmental samples (manure and soil) were collected on the 52 poultry and cattle farms investigated in the study, and animal samples were only collected on poultry farms (Figure 3). An initial visit to poultry farms was scheduled to sample 20 cloacal swabs when the animals were still in the poultry house, a few days before slaughtering. To collect environmental samples, cattle and poultry farms were visited twice, the first visit being one week before manure spreading and the second visit one month after manure spreading. For the botulism-affected farms, the investigation and sample collection were conducted after the botulism outbreak had been diagnosed and management procedures implemented. An epidemiological questionnaire was completed with the farmer during the first visit to collect information about the flock’s health history and manure management practices.

During the first visit, 12 samples of soil and two boot swabs (Sodibox, Nevez, France) were taken in the field selected for manure spreading. Twelve samples of manure and two swabs from the pile were also collected for *C. botulinum* investigation. For 27 farms, one pooled soil sample was also collected for physicochemical analysis. The physicochemical analysis of the soils was added during the course of the study. Consequently, not all 52 plots were analyzed. During the second visit, one month after manure spreading, 12 soil samples and two boot swabs (Sodibox, Nevez, France) on the same field were collected for *C. botulinum* investigation. The choice of a one-month interval between spreading and soil sampling was made to allow sufficient time between spreading and soil sampling to monitor contamination of the field, while ensuring access to the plot without damaging the crops.

Specifics protocols were applied to sample the manure piles and the fields. The manure pile was first divided into three areas, with 30 cm by 30 cm squares in each area; the manure was taken from a depth of 15 cm inside each square, then placed in a bag for homogenization and placed in a 200 mL sampling flask. The two swabs (Sodibox, Nevez, France) were used to wipe each side of the manure pile (one forward and backward swabbing action along the surface of the pile). To collect soil samples, a total of 12 squares of 1 m by 1 m were identified over the surface of the field (approximately one hectare) with stakes. In each square, three soil samples were taken with an auger from a depth of 30 cm and a diameter of 7 cm. These three samples were homogenized in a bucket, and one sample of the soil mixture was placed in a 200 mL sampling flask. Two boot swabs (Sodibox, Nevez, France) were used to do a forward and backward swabbing action along the surface of the field. All the samples were frozen for storage until analysis.

### 5.3. Detection of Clostridium Botulinum in Collected Samples

Swabs and boot swabs were diluted in 250 mL of pre-reduced trypticase-peptone-glucose-yeast extract (TPGY) [34]. A volume of 5 mL of TPGY was added to each cloacal swab. Soil and manure samples (25 g) were ten-fold diluted in pre-reduced TPGY. All the samples were further homogenized for 15 s using a Pulsifier (Microgen, Camberley, UK) and incubated for at least 18 h at 37 °C under anaerobic conditions. After incubation, 1 mL of each enrichment broth was collected. Cells were pelleted by centrifugation, and DNA was extracted using a DNeasy Powersoil Pro kit (Qiagen, Courtaboeuf, France) for swabs, boot swabs and soil samples and using NucleoSpinSoil (Macherey-Nagel, Hoerdt, France) for manure, according to the manufacturer’s instructions. A PCR assay [35] was used to seek the *ntnh* (nontoxic nonhemagglutinin) gene found in the neurotoxin gene cluster of *C. botulinum* group III, that is to say *C. botulinum* C, D, C/D, and D/C. Samples positive for this target were further analyzed to detect genes encoding BoNT-C, D, C/D, and D/C by PCR. Real-time PCR using a Bio-Rad CFX96 thermal cycler (Bio-Rad, Marne-la-Coquette, France) was used to detect the gene target in samples, with a 5 μL DNA template, 10 μL of PerfeCTa qPCR ToughMix (VWR, Briare, France), and primers and probes as described in [36] at a final concentration of 600 nM for primers and 400 nM for probes for each assay. The thermal profile consisted of 5 min at 95 °C, followed by 45 cycles of denaturation for 15 s at 95 °C and annealing/elongation for 30 s at 55 °C. A commercial internal amplification control (QuantiFast Pathogen + IC Kit; Qiagen, Courtaboeuf, France) was used according to the manufacturer’s instructions. A positive control and a negative control were used throughout the entire method (from the enrichment step through to the interpretation of the PCR results) to validate the results and ensure there was no contamination during the analyses. A sample was considered positive when the Ct (cycle threshold) was below 38.

### 5.4. Physicochemical Analysis of Soil

The textural and chemical characteristics of the soil samples were determined according to the methodology developed by the French soil quality monitoring network RMQS, as previously described [31,37]. Soil samples were ground to 2 mm for granulometry analysis and then to 250 μm for chemical analysis (normalized method NF ISO 11464 [38]). A 5-fraction granulometry analysis was performed without decarbonation (NFX 31-107 [39]). Total carbonates were determined by the NF ISO 10693 method [40]. pH in water was determined by the NF ISO 10 390 method [41]. Total organic carbon was measured using ISO 10694 method [42]. Total nitrogen was estimated by the Dumas method (NF ISO 13 878 [43]) and phosphorus by the Joret Hébert method (NFX 31-161 [44]). Cations and CEC Metson were measured by NFX 31-130 method [45]. Other chemicals were analyzed by ICP AES (NFX 31-108) [46].

### 5.5. Statistical Analysis

The data on each flock’s health history, manure management on the farms and *C. botulinum* detection in cloacal swabs, manure, and soil samples were described for each of the six farm categories (clinically unaffected poultry flocks without a history of botulism, clinically unaffected poultry flocks with a history of botulism, clinically unaffected poultry flocks on a mixed farm with an ongoing bovine outbreak, botulism-affected poultry flocks, clinically unaffected bovine farms and botulism-affected bovine farms). The risk of detecting C. botulinum in a manure sample, a soil before spreading and a soil sample after spreading was modelled separately for the three types of samples using a mixed logistic regression model with the farm introduced as a random effect. Type of farm (poultry vs. bovine) and type of sample (swab vs. flask) were introduced as fixed effects in all models. Other factors were tested and kept in the model (forward selection) based on their contribution to the model: ongoing case of bovine botulism on the farm (yes/no), ongoing case of poultry botulism on the farm (yes/no) and history of poultry botulism on the farm (yes/no). For the risk of C. botulinum detection in a soil sample after manure spreading, two other variables were tested: the number of positive soil samples before spreading (0 to 8) and the number of positive manure samples (0 to 14). The models were run using the lme4 [47] package in R (version 4.1.2—“Bird Hippie”) [48]. The model representations were performed with the “plotmodel” function of the package sjPlot 2.9.0 [49]. Statistical relationships between the detection of *C. botulinum* in soil samples and the textural and chemical characteristics of the soils were explored using the Wilcoxon test (catdes function in the factomineR package, version 2.1 [50]).

## Figures and Tables

**Figure 1:**
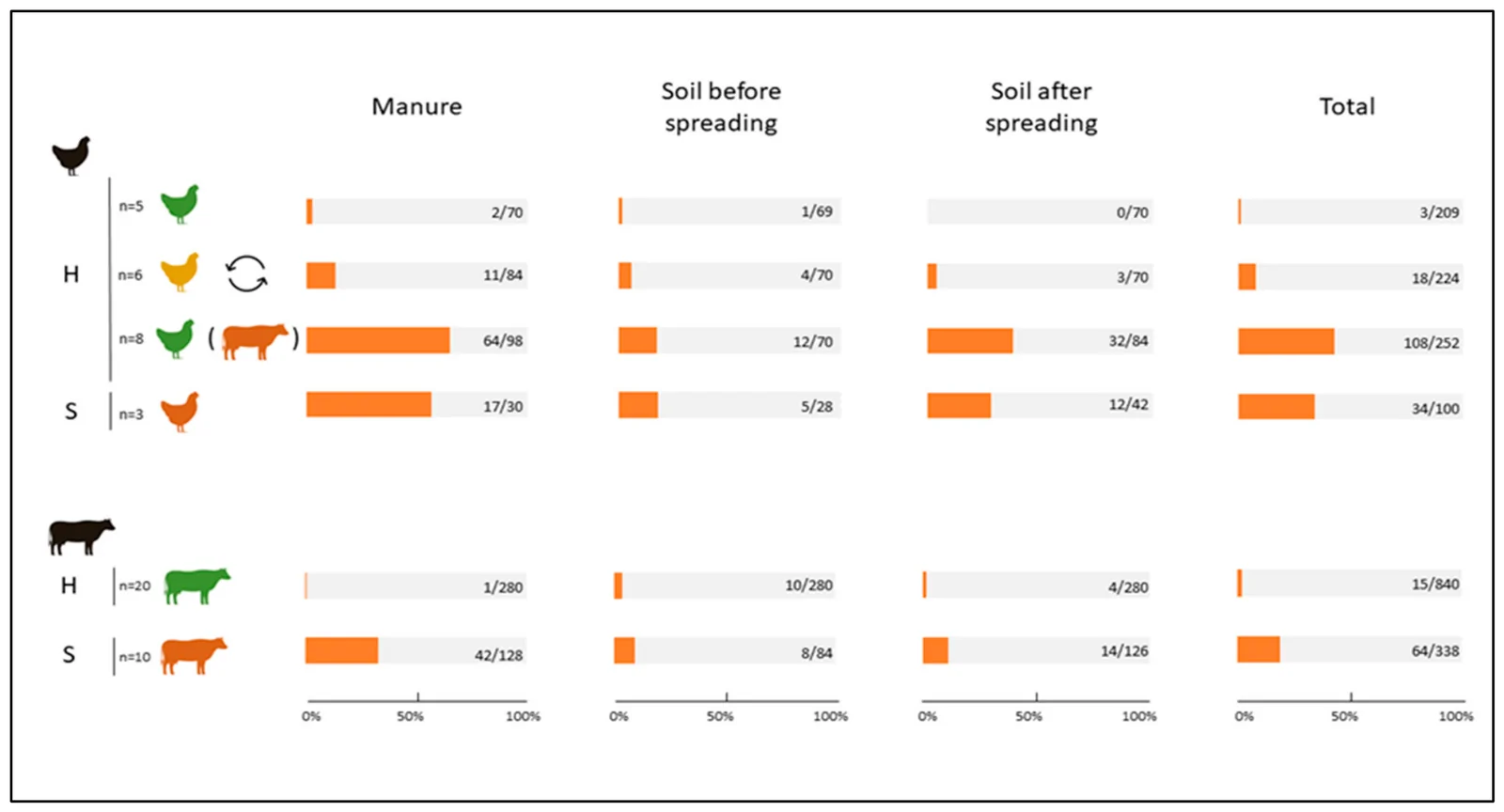
*Clostridium botulinum* (type C, D, C/D, or D/C) detection on poultry farms (n = 22) in the upper part of the figure and on cattle farms (n = 30) in the lower part of the figure. H: Healthy animals with no clinical signs in clinically unaffected farms. S: Sick animals with clinical signs of botulism in clinically affected farms. The percentage of samples for which *C. botulinum* was detected is indicated in orange, and no detection is indicated in light gray. The number of positive samples out of the total number of samples analyzed is indicated on the right-hand side of each graph. In order, from top to bottom: poultry farms with no history of botulism; poultry farms with no clinical signs at the time of sampling but with a history of avian or cattle botulism; poultry flocks on a mixed farm with no clinical signs at the time of sampling and no history of avian botulism but with an ongoing outbreak of bovine botulism; poultry farms with an ongoing outbreak of avian botulism; cattle farms with no history of botulism; cattle farms with an ongoing outbreak of bovine botulism.

**Figure 2:**
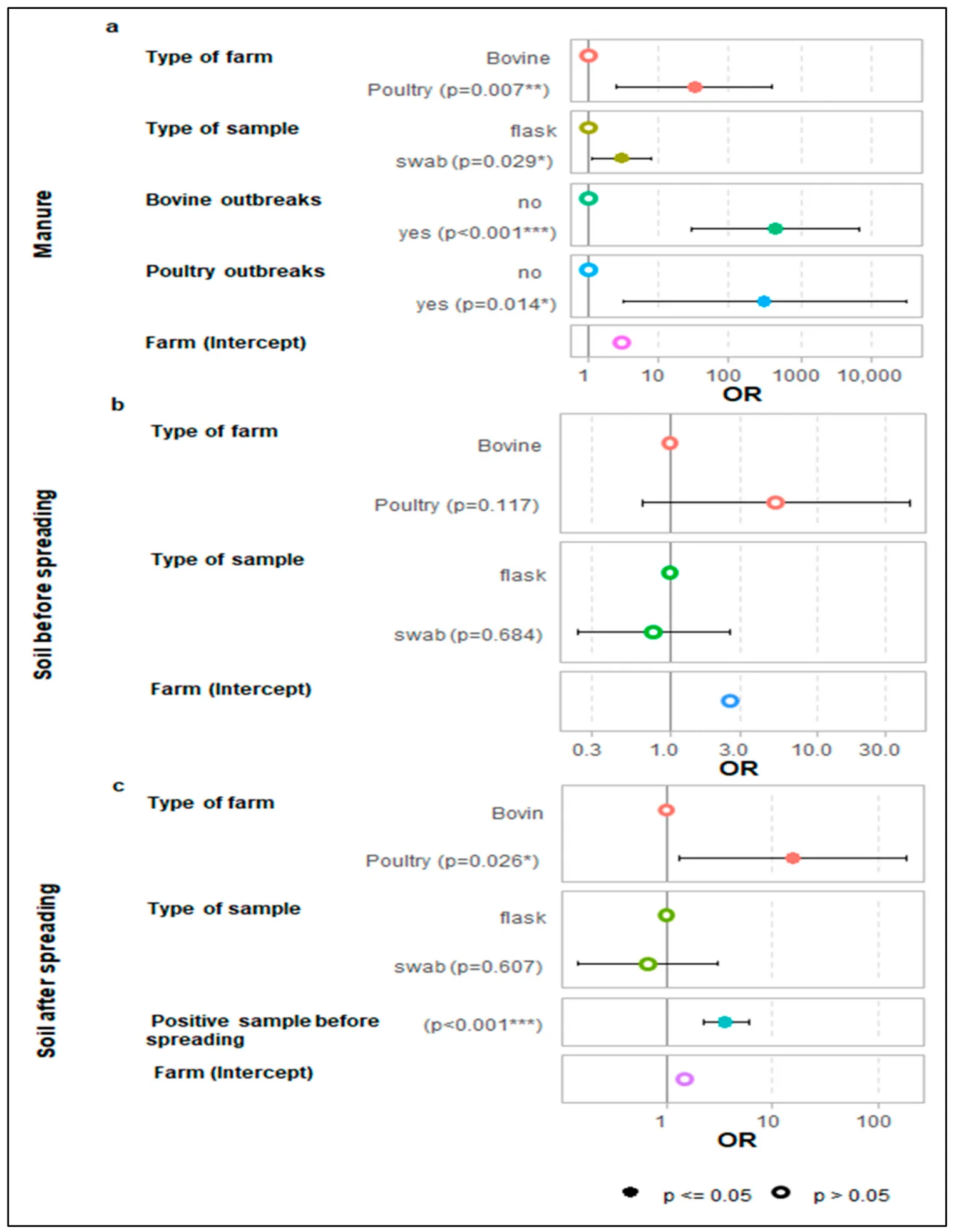
Risk of detecting *C. botulinum* in (**a**) manure samples, (**b**) soil samples before manure spreading and (**c**) soil samples after manure spreading (mixed logistic regression models). Results are shown as odds ratio (OR) with 95% confidence interval. * *p* < 0.05, ** *p* < 0.01, *** *p* < 0.001.

**Figure 3:**
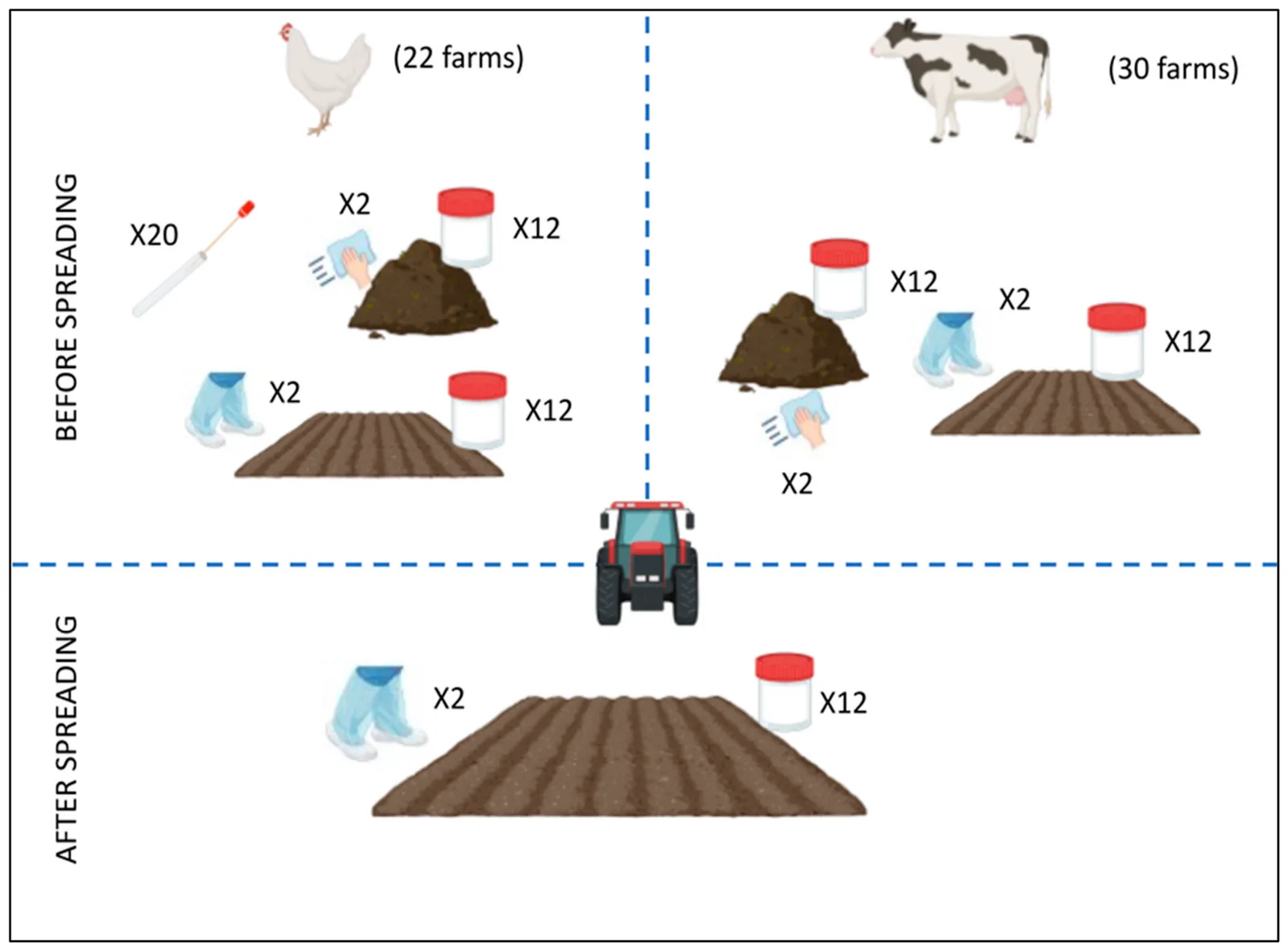
Protocol of sampling plan on the 22 poultry farms and 30 cattle farms The number of samples collected per category is shown next to each sample’s symbol (X = multiplied by) (created in BioRender. Biorender, H. (2026) https://BioRender.com/wnkhsj8).

**Table 1:** Description of poultry farms investigated and manure management (n = 22).

	Clinically Unaffected Poultry Flocks with No History of Botulism (n = 5)	Clinically Unaffected Poultry Flocks with a History of Botulism (n = 6)	Clinically Unaffected Poultry Flocks with an Ongoing Bovine Outbreak (n = 8)	Botulism-Affected Poultry Flocks (n = 3)
Poultry production	Turkeys (4) Broilers (1)	Turkeys (3) Broilers (3)	Broilers (6) Hens (1) Turkeys (1)	Turkeys (3)
Manure storage time	Average storage time (4): 7.5 weeks (from 1 to 12), no storage (1)	Average storage time (6): 17.2 weeks (from 6 to 40)	Average storage time (7): 15 weeks (from 1 to 40), no storage (1)	Storage for 3 months (1), no storage (2)
Manure spreading: depth of burial	Average depth of burial (4): 20 cm (10 to 30 cm) MD (1)	Average depth of burial (4): 21 cm (10 to 40 cm), MD (1) and field not investigated (1)	Average depth of burial (6): 22.5 cm (15 to 30 cm), no burial (1) and field not investigated (1)	Depth of burial (3): 30 cm

MD: Missing data.

**Table 2:** Description of bovine farms investigated and manure management (n = 30).

	Clinically Unaffected Bovine Farms (n = 20)	Botulism-Affected Bovine Farms (n = 10)
Bovine production	Dairy farms (16) Dairy and beef farms (3) Beef farm (1)	Dairy farms (8) Dairy and beef farms (2)
Manure storage time	Average storage time (20): 14 weeks (from 3 to 40)	Average storage time (7): 20.5 weeks (from 10 to 30), no storage (1) and MD (2). Lime in 3 manure piles.
Manure spreading: depth of burial	Average depth of burial (17): 16,7 cm (10 to 30 cm) and no burial (3)	Average depth of burial (7): 23.5 cm (15 to 30 cm), no burial (1), field not investigated (1) and MD (1)

MD: Missing data.

## Data Availability

The original contributions presented in this study are included in the article/Supplementary Materials. Further inquiries can be directed to the corresponding author.

## Supplementary Materials

The following supporting information can be downloaded at: https://www.mdpi.com/article/10.3390/toxins18100406/s1, Supplementary Data S1: Description of poultry farms investigated and manure management (n = 22); Supplementary Data S2: Description of bovine farms investigated and manure management (n = 30); Supplementary Data S3: (a). Clostridium botulinum detection (type C, D, C/D, or D/C) on clinically unaffected poultry farms without a history of botulism (n = 5). (b). Clostridium botulinum detection (type C, D, C/D, or D/C) on clinically unaffected poultry farms with a history of botulism (n = 6). (c). Clostridium botulinum detection (type C, D, C/D or D/C) on clinically unaffected poultry farms with an ongoing bovine outbreak (n = 8). (d). Clostridium botulinum detection (type C, D, C/D or D/C) on affected poultry farms (n = 3); Supplementary Data S4: (a). Clostridium botulinum detection (type C, D, C/D, or D/C) on clinically unaffected bovine farms (n = 20). (b). Clostridium botulinum detection (type C, D, C/D, or D/C) on botulism-affected bovine farms (n = 10); Supplementary Data S5: Soil characteristics.
