# Supplementary material for: Monitoring of Clostridium botulinum in Poultry and Cattle Manure During Spreading Operations
Source: Toxins (Basel). 2026 Sep 22;18(10):406. doi: 10.3390/toxins18100406 (PMC13709867; doi:10.3390/toxins18100406)
Supplement: Supplementary file 1 [file toxins-18-00406-s001.zip › toxins-4531686-supplementary.pdf]

Supplementary Materials: Monitoring of Clostridium Botulinum in Poultry and Cattle Manure During Spreading Operations

Rozenn Souillard, Sandra Rouxel, Virginie Dorenlor, Grégoire Kuntz, Félix Mahé, Typhaine Poezevara, Florent Eono, Amandine Avouac, Stéphane Kerphérique, Léa Jambou, Eloïse Cailton, Mathys Gouet, Sébas-tien Solanas, Loïc Balaine, Adeline Huneau-Salaün, Marianne Chemaly, Sophie Le Bouquin, Alain Hartmann and Caroline Le Maréchal

Supplementary Data S1. Description of poultry farms investigated and manure management (n=22)

	**No.**	**Poultry production (mixed farm poultry/cattle)**	**Manure storage time**	**Manure spreading: burial depth**	**Spreading over the field for the past 2 years**	**Previous use of the field**
**Clinically unaffected poultry flocks without a history of botulism (n=5)**	VT10	Broilers (no mixed farm)	8 weeks	30 cm	Poultry manure	Plant cover
	VT12	Turkeys (no mixed farm)	12 weeks	10 cm	Poultry manure	Cereal crop
	VT5	Turkeys (mixed farm)	1 week	10 cm	Poultry and bovine manure	Cereal crop
	VT7	Turkeys (no mixed farm)	9 weeks	MD	Poultry manure	Cereal crop
	VT9**a**	Turkeys (mixed farm)	No storage	30 cm	Poultry manure	Cereal crop
**Clinically unaffected poultry flocks with a history of botulism (n=6)** (type detected in previous botulism events)	VT1**b** (Poultry CD)	Turkeys (mixed farm)	40 weeks	-	-	-
	VT18 (Poultry CD)	Turkeys (no mixed farm)	16 weeks	40 cm	Poultry manure	Cereal crop
	VT19 (Bovine type = MD)	Turkeys (mixed farm)	13 weeks	15 cm	Poultry manure	Cereal crop
	VT3 (Bovine type = MD)	Broilers (mixed farm)	6 weeks	10 cm	Poultry and bovine manure	Cereal crop
	VT6 (Bovine D or D/C)	Broilers (mixed farm)	17 weeks	MD	Poultry manure	Cereal crop
	VT8 (Poultry type = MD)	Broilers (mixed farm)	11 weeks	20 cm	Poultry manure	Cereal crop
**Clinically unaffected poultry flocks with an ongoing bovine outbreak (n=8)** (bovine botulism type/No. of bovine farm also investigated in the study)	VF-BC10**c** (type C/BC10)	Hens (mixed farm)	20 weeks	No burial	Poultry and bovine manure	Cereal crop
	VT13 (type DC)	Broilers (mixed farm)	1 week	30 cm	No spreading	Bovine pasture
	VT14 (type DC/BC8)	Broilers (mixed farm)	21 weeks	15 cm	No spreading	Cereal crop
	VT16 (type DC/BC7)	Turkeys (mixed farm)	1 week	30 cm	Poultry and bovine manure	Plant cover
	VT17**d** (type DC/BC9)	Broilers (mixed farm)	No storage	15 cm	No spreading	Cereal crop
	VT2 (type DC)	Broilers (mixed farm)	40 weeks	15 cm	bovine manure	Cereal crop
	VT20**e** (type DC)	Broilers (mixed farm)	12 weeks	-	-	-
	VT21 (type DC)	Broilers (mixed farm)	10 weeks	30 cm	Poultry and bovine manure	Cereal crop
**Botulism-affected poultry flocks (n=3)**	VC1**f**	Turkeys type C/D (farm = MD)	No storage	30 cm	MD	MD
	VC2**g**	Turkeys type C/D (mixed farm)	No storage	30 cm	Poultry and bovine manure	Cereal crop

	VC3	Turkeys type D/C (farm = MD)	12 weeks	30 cm	MD	MD

[a]VT9: no manure storage: manure sampled in the poultry house. [b]VT1 It was not possible to investigate the field. [c]VF-BC10: hens' manure spread with the bovine manure of the ongoing outbreak on the farm BC10. [d]VT17 no manure storage: manure sampled in the poultry house. [e]VT20 it was not possible to investigate the field. [f]VC1 no manure storage: manure sampled in the poultry house. [g]VC2 no manure storage: manure sampled in the poultry house.

MD: missing data

Mixed farm: farms with both poultry and cattle

Supplementary Data S2. Description of bovine farms investigated and manure management (n=30)

	No.	Bovine production (mixed farm poultry/cattle)	Manure storage time	Manure spreading: burial depth	Manure spread over field in previous 2 years	Previous use of the field
**Clinically**	**BT1**	Dairy (not mixed farm)	20 weeks	15 cm	Bovine manure	Cereal crop
**unaffected**	**BT10**	Dairy (not mixed farm)	8 weeks	20 cm	Bovine manure	Cereal crop
**bovine**	**BT11**	Dairy (not mixed farm)	24 weeks	15 cm	No spreading	Cereal crop
**farms**	**BT12**	Dairy (not mixed farm)	8 weeks	15 cm	Bovine manure	Cereal crop
**(n=20)**	**BT13**	Dairy and beef (not mixed farm)	32 weeks	20 cm	Bovine and pig manure	Cereal crop
	**BT14**	Dairy (not mixed farm)	40 weeks	15 cm	Bovine manure	Cereal crop
	**BT15**	Dairy (mixed farm)	12 weeks	20 cm	Poultry and bovine manure	Cereal crop
	**BT16**	Dairy (not mixed farm)	8 weeks	No burial	Bovine manure	Bovine pasture
	**BT17**	Dairy (not mixed farm)	7 weeks	No burial	Bovine manure	Bovine pasture
	**BT18**	Dairy and beef (not mixed farm)	8 weeks	No burial	Bovine manure	Bovine pasture
	**BT19**	Dairy (not mixed farm)	4 weeks	15 cm	Bovine and pig manure	Cereal crop
	**BT2**	Dairy (not mixed farm)	6 weeks	30 cm	Bovine manure	Plant cover
	**BT20**	Beef (not mixed farm)	22 weeks	20 cm	No spreading	Plant cover
	**BT3**	Dairy (not mixed farm)	20 weeks	20 cm	Pig manure	Plant cover
	**BT4**	Dairy (not mixed farm)	3 weeks	10 cm	Bovine manure	Cereal crop
	**BT5**	Dairy (mixed farm)	3 weeks	25 cm	Bovine manure	Cereal crop
	**BT6**	Dairy and beef (not mixed farm)	18 weeks	15 cm	Bovine manure	Cereal crop
	**BT7**	Dairy (not mixed farm)	12 weeks	10 cm	Poultry and bovine manure	Cereal crop
	**BT8**	Dairy (not mixed farm)	6 weeks	10 cm	Bovine manure	Cereal crop
	**BT9**	Dairy (not mixed farm)	20 weeks	10 cm	Bovine manure	Plant cover
**Affected**	**BC0**	Dairy and beef type D/C (mixed farm)	10 weeks with lime	25 cm with lime	No spreading	Cereal crop
**bovine**	**BC1a**	Dairy type D/C (mixed farm)	MD	30 cm	Bovine and poultry manure	Cereal crop
**farms**	**BC10b**	Dairy type C (mixed farm VF-BC10)	20 weeks	No burial	Bovine and poultry manure	Cereal crop
**(n=10)** with	**BC11c**	Dairy type D/C (not mixed farm)	No storage	25 cm	No spreading	Cereal crop
No. of	**BC2d**	Dairy and beef type D/C (not mixed farm)	28 weeks with lime	-	-	-
poultry	**BC3**	Dairy (MD type and mixed farm)	30 weeks	MD	MD	MD
flock also	**BC6**	Dairy type D/C (not mixed farm)	20 weeks with lime	20 cm	MD	Cereal crop
investigated	**BC7**	Dairy type D/C (mixed farm VT16)	16 weeks	30 cm	Bovine manure	Cereal crop
in the study	**BC8e**	Dairy type D/C (mixed farm VT14)	20 weeks	15 cm	Poultry and bovine manure	Cereal crop
	**BC9f**	Dairy type D/C (mixed farm VT17)	MD	20 cm	Bovine manure	Cereal crop

[a]BC1 liquid manure for this farm. [b]BC10: bovine manure spread with the hens' manure VF-BC10. [c]BC11 it was not possible to sample manure or field before spreading. [d]BC2 it was not possible to investigate the field. eBC8 it was not possible to sample the field before spreading. [f]BC9 it was not possible to sample the manure or soil before spreading.

MD: missing data

Mixed farms: farms with both cattle and poultry

Supplementary Data S3 (a). *Clostridium botulinum* detection (type C, D, C/D, or D/C) on clinically unaffected poultry farms without a history of botulism (n=5)

			Detection	VT10	VT12	VT5	VT7b	VT9a	Total	% positive samples	Nb. of positive farms
Environmental samples	Manure	Swab	Not detected	2	2	2	2	2	10	**2.8% (2/70)**	**2/5**
		Flask	Detected			1		1	2		
			Not detected	12	12	11	12	11	58		
	Soil before spreading	Swab	Not detected	2	2	2	2	2	10	**1.4% (1/69)**	**1/5**
		Flask	Detected				1		1		
			Not detected	12	12	12	10	12	58		
	Soil after spreading	Swab	Not detected	2	2	2	2	2	10	**0% (0/70)**	**0/5**
		Flask	Not detected	12	12	12	12	12	60		
	**Total**			**42**	**42**	**42**	**41**	**42**	**209**	**1.4% (3/209)**	**3/5**
Animal samples	Cloacal swabs		Detected			1			1	**1% (1/100)**	**1/5**
			Not detected	20	20	19	20	20	99		

[a] VT9: no manure storage: manure sampled in the poultry house [b]VT7: 11 flasks of soil collected before spreading (instead of 12)

Supplementary Data S3 (b). *Clostridium botulinum* detection (type C, D, C/D, or D/C) on clinically unaffected poultry farms with a history of botulism (n=6)

			Detection	VT1a	VT18	VT19	VT3	VT6	VT8	Total	% positive samples	Nb. of positive farms
Environmental samples	Manure	Swab	Detected		1	1				2	**13.1% (11/84)**	**3/6**
			Not detected	2	1	1	2	2	2	10		
		Flask	Detected		6	1		2		9		
			Not detected	12	6	11	12	10	12	63		

	Soil before spreading	Swab	Not detected		2	2	2	2	2	10	**5.7% (4/70)**	**3/5**
		Flask	Detected		2	1		1		4		
			Not detected		10	11	12	11	12	56		
	Soil after spreading	Swab	Detected					1		1	**4.3% (3/70)**	**2/5**
			Not detected		2	2	2	1	2	9		
		Flask	Detected		2					2		
			Not detected		10	12	12	12	12	58		
	**Total**			**14**	**42**	**42**	**42**	**42**	**42**	**224**	**8% (18/224)**	**3/6**
Animal samples	Cloacal swabs		Not detected	20	20	20	20	20	20	120	**0% (0/120)**	**0/6**

[a]VT1 It was not possible to investigate the field

Supplementary Data S3 (c). *Clostridium botulinum* detection (type C, D, C/D or D/C) on clinically unaffected poultry farms with an ongoing bovine outbreak (n=8)

				**VF-BC10a**	**VT13**	**VT14b**	**VT16c**	**VT17d**	**VT2**	**VT20e**	**VT21**	**Total**	**% positive samples**	**Nb. of positive farms**
Environmental samples	Manure	Swab	Detected		2	2		2	2	2		10	**65.3% (64/98)**	**5/7**
			Not detected	2							2	4		
		Flask	Detected		11	12		12	12	7		54		
			Not detected	12	1					5	12	30		
	Soil before spreading	Swab	Detected					1	1			2	**17.1% (12/70)**	**3/5**
			Not detected		2		2	1	1		2	8		
		Flask	Detected					2	4		4	10		
			Not detected		12		12	10	8		8	50		
	Soil after spreading	Swab	Detected						2		1	3	**38.1% (32/84)**	**3/6**
			Not detected		2	2	2	2			1	9		
		Flask	Detected					8	10		11	29		
			Not detected		12	12	12	4	2		1	43		
	**Total**			**14**	**42**	**28**	**28**	**42**	**42**	**14**	**42**	**252**	**42.8% (108/252)**	**6/8**
Animal samples	Cloacal swabs		Detected		11				3			14	**23.3% (14/60)**	**2/3**
			Not detected		9				17		20	46		

[a]VF-BC10: hens' manure spread with the bovine manure of the ongoing outbreak on the farm BC10, the soil analysis results are presented in Supp. Data 4 (b), botulism-affected bovine farms. [b]VT14 it was not possible to sample the soil before spreading. [c]VT16 it was not possible to sample the manure. [d]VT17 no manure storage: manure sampled in the poultry house. [e]VT20 it was not possible to investigate the field.

Supplementary Data S3 (d). *Clostridium botulinum* detection (type C, D, C/D or D/C) on affected poultry farms (n=3)

			**Detection**	**VC1[a]**	**VC2[b]**	**VC3**	**Total**	**% positive samples**	**Nb. of positive farms**
Environmental samples	Manure	Swab	Detected	2	2	2	6	**56.7% (17/30)**	**3/3**
		Flask	Detected		2	9	11		
			Not detected		10	3	13		
	Soil before spreading	Swab	Not detected		2	2	4	**17.8% (5/28)**	**1/2**
		Flask	Detected			5	5		
			Not detected		12	7	19		
	Soil after spreading	Swab	Detected			1	1	**28.6% (12/42)**	**2/3**
			Not detected	2	2	1	5		
		Flask	Detected	11			11		
			Not detected	1	12	12	25		
	**Total**			**16**	**42**	**42**	**100**	**34% (34/100)**	**3/3**
Animal samples	Cloacal swabs		Detected	15		5	20	**50% (20/40)**	**2/2**
			Not detected	5		15	20		

[a]VC1 no manure storage: manure sampled in the poultry house (only 2 swabs) and it was not possible to sample soil before spreading.

[b]VC2 no manure storage: manure sampled in the poultry house

Supplementary Data S4 (a). *Clostridium botulinum* detection (type C, D, C/D, or D/C) on clinically unaffected bovine farms (n=20)

	Sample	Detection	BT1	BT10	BT11	BT12	BT13	BT14	BT15	BT16	BT17	BT18	BT19	BT2	BT20	BT3	BT4	BT5	BT6	BT7	BT8	BT9	Total	% positive samples	Nb. of positive farms
Manure	Swab	Detected								1													1	**0.3% (1/280)**	**1/20**
		Not detected	2	2	2	2	2	2	2	1	2	2	2	2	2	2	2	2	2	2	2	2	39		
	Flask	Not detected	12	12	12	12	12	12	12	12	12	12	12	12	12	12	12	12	12	12	12	12	240		
Soil before spreading	Swab	Detected													1				1				2	**3.6% (10/280)**	**4/20**
		Not detected	2	2	2	2	2	2	2	2	2	2	2	2	1	2	2	2	1	2	2	2	38		
	Flask	Detected	1								2				5								8		
		Not detected	11	12	12	12	12	12	12	12	10	12	12	12	7	12	12	12	12	12	12	12	232		
Soil after spreading	Swab	Not detected	2	2	2	2	2	2	2	2	2	2	2	2	2	2	2	2	2	2	2	2	40	**1.4% (4/280)**	**2/20**
	Flask	Detected									1				3								4		
		Not detected	12	12	12	12	12	12	12	12	11	12	12	12	9	12	12	12	12	12	12	12	236		
	**Total**		**42**	**42**	**42**	**42**	**42**	**42**	**42**	**42**	**42**	**42**	**42**	**42**	**42**	**42**	**42**	**42**	**42**	**42**	**42**	**42**	**840**	**1.8% (15/840)**	**5/20**

Supplementary Data S4 (b). *Clostridium botulinum* detection (type C, D, C/D, or D/C) on botulism-affected bovine farms (n=10)

	**Sample**	**Detection**	**BC0**	**BC1a**	**BC10b**	**BC11c**	**BC2d**	**BC3**	**BC6**	**BC7**	**BC8e**	**BC9f**	**Total**	**% positive samples**	**Nb. of positive farms**
Manure	Swab	Detected	1				1			2	2		6	**32.8% (42/128)**	**5/8**
		Not detected	1	2	2		1	2	2				10		
	Flask*	Detected	4	12			9			1	10		36		
		Not detected	8		12		11	14	14	13	4		76		
Soil before spreading	Swab	Not detected	2	2	2			2	2	2			12	**9.5% (8/84)**	**1/6**
	Flask	Detected	8										8		
		Not interpretable (PCR inhibitors)		3									3		
		Not detected	4	9	12			12	12	12			61		
Soil after spreading	Swab	Detected										1	1	**11.1% (14/126)**	**2/9**
		Not detected	2	2	2	2		2	2	2	2	1	17		
	Flask	Detected	12									1	13		
		Not detected		12	12	12		12	12	12	12	11	95		
**Total**			**42**	**42**	**42**	**14**	**22**	**44**	**44**	**44**	**30**	**14**	**338**	**18.9% (64/338)**	**6/10**

[a]BC1: liquid manure for this farm. [b]BC10: cattle manure spread with the hens' manure VF-BC10. [c]BC11: it was not possible to sample the manure or field before spreading. [d]BC2: it was not possible to sample the field. [e]BC8: it was not possible to sample the field before spreading. [f]BC9: it was not possible to sample the manure or soil before spreading.

*12 flasks of manure except for farm BC2 (n=20), BC3, BC6, BC7, and BC8 (n=14).

Supplementary Data S5. Soil characteristics

Textural and chemical characteristics of soils analyzed for *C. botulinum* detection (France, 2020-2023) (n=27)

parameter	mean	sd	min	med	max	method	unit	detection threshold
clay (< 2µm)	18	7.7	7.1	18.3	38.1	NF X 31-107	g/kg	
silt (2-20 µm)	22.4	10.5	3.1	23.6	43.7	NF X 31-107	g/kg	
silt (20-50 µm)	25.6	12.1	4.1	22.6	47.5	NF X 31-107	g/kg	
sand (50-200 µm)	16.5	9.3	6.0	14.4	43.8	NF X 31-107	g/kg	
sand (200 - 2000 µm)	34.0	19.7	7.1	25.4	74.5	NF X 31-107	g/kg	
cation exchange capacity	11.1	6.5	3.1	8.5	28.7	NF X 31-130	cmol+/kg	1
exchangeable calcium	7.0	8.0	0.3	6	28.7	NF X 31-130	cmol+/kg	0,01
exchangeable potassium	0.5	0.3	0.2	0.5	1.5	NF X 31-130	cmol+/kg	0,02
exchangeable magnesium	0.6	0.5	0.0	0.6	1.8	NF X 31-130	cmol+/kg	0,005
exchangeable sodium	0.1	0.0	0.0	0.1	0.2	NF X 31-130	cmol+/kg	0,005
exchangeable manganese	0.0	0.0	0.0	0.0	0.1	NF X 31-130	cmol+/kg	0,005
exchangeable aluminum	0.1	0.1	0.0	0.0	0.3	NF X 31-130	cmol+/kg	0,02
exchangeable iron	0.0	0.0	0.0	0.0	0.0	NF X 31-130	cmol+/kg	0,005
available phosphorus	0.1	0.1	0.0	0.1	0.3	Olsen method	g/kg	0,005
total limestone	14.9	41.6	1.0	5.0	213	NF ISO 10693	g/kg	1
pH	6.3	0.9	5.0	6.0	8.0	NF ISO 10390		
humidity	15.5	6.6	7.5	13.3	29.2	NF ISO 11465	g/kg	0
organic carbon	22.5	8.3	10.6	21.9	43.8	dry combustion	g/kg	0,05
total nitrogen	2.5	0.9	1.1	2.6	4.5	dry combustion	g/kg	0,02
organic matter	38.9	14.4	18.3	37.9	75.7	dry combustion	g/kg	0,086
